# Frailty over the rainbow: a cross-sectional study of LGBT+ adults aged over 50 years

**DOI:** 10.31744/einstein_journal/2024AO0937

**Published:** 2024-11-26

**Authors:** Leonardo Rabelo de Melo, Milton Roberto Furst Crenitte, Richard Green, Wilson Jacob, Thiago Junqueira Avelino-Silva

**Affiliations:** 1 Universidade de São Paulo Faculdade de Medicina Hospital das Clínicas São Paulo SP Brazil Division of Geriatrics, Hospital das Clínicas, Faculdade de Medicina, Universidade de São Paulo, São Paulo, SP, Brazil.; 2 Universidade de São Caetano do Sul Faculdade de Medicina São Caetano do Sul SP Brazil Faculdade de Medicina, Universidade de São Caetano do Sul, São Caetano do Sul, SP, Brazil.; 3 Universidade de São Paulo Faculdade de Medicina Hospital das Clínicas São Paulo SP Brazil Laboratório de Investigação Médica em Envelhecimento (LIM-66), Division of Geriatrics, Hospital das Clínicas, Faculdade de Medicina, Universidade de São Paulo, São Paulo, SP, Brazil.; 4 University of Surrey School of Health Sciences and Surrey Institute for People-Centred AI Surrey United Kingdom School of Health Sciences and Surrey Institute for People-Centred AI, University of Surrey, Guildford, Surrey, United Kingdom.; 5 Hospital Israelita Albert Einstein Faculdade Israelita de Ciências da Saúde Albert Einstein São Paulo SP Brazil Faculdade Israelita de Ciências da Saúde Albert Einstein, Hospital Israelita Albert Einstein, São Paulo, SP, Brazil.

**Keywords:** Frailty, Middle aged, Aged, Minority health, Sexual and gender minorities, Survey and questionnaire

## Abstract

**Objective:**

To investigate differences in the prevalence of frailty between LGBT+ and non-LGBT+ older adults.

**Methods:**

A cross-sectional study involving Brazilians aged 50 and over was performed. The participants were invited to participate in an anonymous online survey between August 2019 and January 2020. The survey was widely distributed in neighborhood associations, day centers, non-governmental organizations, and social media. Those who identified as homosexual, bisexual, pansexual, non-heterosexual, transgender, *travesti*, or non-binary were grouped as LGBT+. Participants who were both cisgender and heterosexual were categorized as non-LGBT+. Frailty was measured using the FRAIL scale, and the participants were categorized as robust, prefrail, or frail. Generalized ordered logistic models were used to examine the adjusted association between LGBT+ status and frailty according to age and sex.

**Results:**

The study sample included 6,693 participants with a median age of 60 years. Overall, 1,332 patients were LGBT+ (19.9%), and 5,361 were non-LGBT+ (80.1%). Pre-frailty or frailty was observed in 656 (49%) LGBT+ participants and 2,460 (46%) non-LGBT+ participants (p=0.03). Multivariate analyses showed that being LGBT+ was independently associated with frailty in female participants aged ≥50 years (OR=1.52, 95%CI=1.08-2.13, p=0.02) and male participants aged ≥60 years (OR=2.83, 95%CI=1.41-5.69, p=0.004). Non-cisgender participants were also more likely to be frail than cisgender participants (OR=2.21, 95%CI=1.42-3.42, p<0.001).

**Conclusion:**

LGBT+ status was independently associated with frailty in female adults aged >50 years and males aged > 60 years. More research in this area and inclusive government policies are needed to promote the healthy aging of the LGBT+ population.

## In Brief

Frailty is a geriatric syndrome associated with negative outcomes such as functional loss, hospitalization, and death. In a novel study conducted in Brazil on people aged 50 years and older, the authors demonstrate that being an LGBT+ older adult is independently associated with frailty. Specifically, being a trans person is independently associated with frailty compared to being cisgender.

## Highlights

■ The FRAIL scale is a validated instrument for the diagnosis of frailty syndrome.
■ LGBT+ people are subject to different stressors throughout their lives, which can affect their health.
■ Being an LGBT+ man aged ≥60 and an LGBT woman aged ≥50 was independently associated with frailty.

## Graphical abstract

**Figure:**
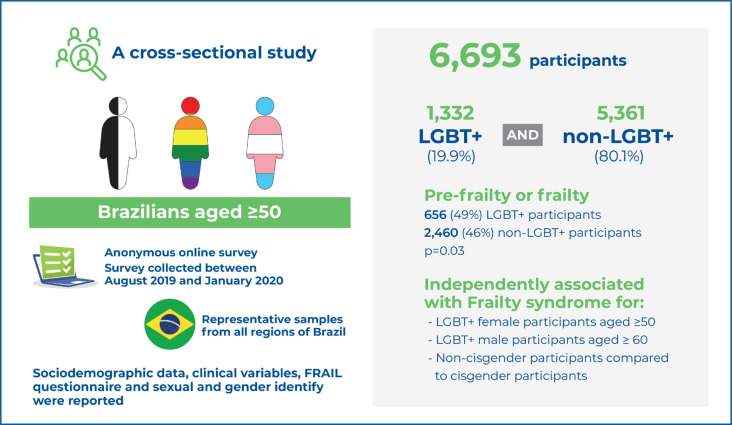

## INTRODUCTION

Despite recent sociocultural advances, Lesbian, Gay, Bisexual, and Transgender (LGBT+) people are frequently stigmatized and marginalized as a group in various social settings including in healthcare settings. Furthermore, recent research has found that many LGBT+ people feel discriminated against in healthcare institutions and often avoid disclosing their sexuality to healthcare providers.^(1)^ This creates the risk of LGBT+ individuals avoiding seeking medical assistance out of fear of discrimination and due to the absence of confidence in the system. Moreover, even if they overcome their fears and seek medical attention (*e.g.*, in emergencies), evidence suggests that the LGBT+ community has a higher risk of inadequate follow-up and adverse outcomes.^(2,3)^

Recently, the concept of "minority stress" has gained attention. The central premise is that minority groups, such as the LGBT+ community, frequently face chronic stressors (*e.g*., non-acceptance, marginalization, internalized homophobia, victimization, and various types of violence) that directly impact their mental health.^(4)^ Thus, unsurprisingly, LGBT+ individuals would have distinctive health risks and complications compared with the heterosexual cisgender population.^(5)^ Numerous studies support this conclusion, reporting higher rates of depression, self-harm, suicide, addiction, obesity, hypertension, and diabetes among gay and bisexual individuals.^(6–8)^ These findings have prompted researchers to speculate that belonging to a sexual or gender minority may be associated with unhealthier aging processes. It may also suggest that models primarily focused on heterosexual aging are inadequate to understand the peculiarities of aging experiences in those with non-conforming sexual orientations.^(9)^

In recent years, research on aging has increasingly focused on the concept of frailty and its impact on older adults.^(10,11)^ Frailty is a measure of physiological reserve, and it is widely acknowledged that frail older adults are more vulnerable to adverse outcomes, such as functional loss, physical limitation, falls, fractures, hospitalization, and mortality.^(12)^ The association between frailty and adverse outcomes makes the syndrome a vital issue for geriatric care. Moreover, frailty is an increasingly common condition, and it is estimated that in Brazil, Europe, and the United States, frailty affects approximately 10% of the population aged over 50 years and 15% of those aged 65 years or older.^(13)^

However, little is known about frailty among older LGBT+ populations. Given that this group is more likely to suffer from depression and other chronic conditions as well as being less inclined to seek medical assistance, it is reasonable to assume that they are also at a higher risk of frailty.^(11,14)^ In our previous studies, we found a higher incidence of loneliness, fear of dying alone, and fear of dying in pain in LGBT+ subjects than in non-LGBT+ subjects.^(15)^ These studies also showed worse experiences when using health services and increased difficulty in accessing these services for the LGBT+ population.^(16)^ Therefore, in this study, we verify the hypothesis that LGBT+ older adults have a higher prevalence of frailty than non-LGBT+ older adults. We believe that the insights gained from this study will expand the understanding of the health needs and difficulties of LGBT+ individuals to inform improvements in the healthcare system.

## OBJECTIVE

To investigate the prevalence of frailty in older LGBT+ adults and compare it with a corresponding heterosexual cisgender sample.

## METHODS

### Study design and population

In this study, we conducted an online cross-sectional survey with Brazilians aged 50 and over. The age cutoff was selected based on previous research investigating LGBT+ health.^(7,8)^ Participants were invited to complete an online survey created and managed using Research Electronic Data Capture (REDCap) resources. The study was promoted by medical associations, patient organizations, neighborhood associations, day centers, and non-governmental organizations. We also distributed the questionnaire's web link on social networks such as Instagram, Facebook, WhatsApp, and YouTube and encouraged participants to forward the information to their social groups, using "snowball sampling" recruitment strategies.^(17)^

We included eligible candidates who consented to participate in the study and provided complete questionnaire responses.

### Data collection

Participants completed a thorough questionnaire detailing their sociodemographic and clinical characteristics, healthcare service utilization, and previous discriminatory and victimization experiences.

Our main independent variables were gender (cisgender male, cisgender female, transgender male, transgender female, *travesti*, non-binary, other) and sexual orientation (heterosexual, homosexual, bisexual, pansexual, asexual, other). For analysis, we created an additional variable grouping: non-LGBT+ (cisgender male, cisgender female, heterosexual) and LGBT+ (transgender male, transgender female, *travesti*, non-binary, homosexual, bisexual, pansexual, other). *Travesti* is a transfeminine person who identifies with a *travesti* gender identity, that has been marginalized throughout history. It is predominantly a Brazilian identity construction but is also found in other Latin American and European countries.^(18)^

Our primary dependent variable was frailty status as defined by the FRAIL scale,^(19)^ which assigns one point to each of the following five attributes: tiredness ("Do you feel fatigued?"); resistance ("Can you climb one flight of stairs?"); ambulation ("Can you walk one block?"); illness ("Do you have more than five illnesses?"); weight loss ("Have you lost more than five percent of your weight in the last six months?"), with the following classification: 0 = robust, 1-2 points = pre-frail, 3 or more points = frail.^(20,21)^

### Statistical analysis

We described our data using central tendency and dispersion measures, counts, and proportions. We compared LGBT+ and non-LGBT+ participants using contingency tables, χ^2^ tests, Fisher's exact tests, Student's *t*-tests, or Wilcoxon's rank-sum tests as appropriate.

We examined generalized ordered logistic models to examine the adjusted association between belonging to an LGBT+ group and frailty status and stratified our analyses according to biological sex (male, female) and age (<60, ≥60). Since the LGBT+ population is not affected uniformly by the same stressors and because past research suggests that transgender individuals experience poorer socioeconomic conditions and greater prejudice than other groups among the LGBT+ community,^(22,23)^ we performed a sensitivity analysis modifying our primary independent variable to group transgender people, non-binary genders, and other genders as non-cisgenders. All the models were adjusted for age, race/ethnicity, public healthcare system utilization, hypertension, diabetes, cancer, obstructive lung disease, asthma, coronary disease, heart failure, cerebrovascular disease, and chronic kidney disease. These variables were self-reported by the participants. We reported the adjusted odds ratios (OR) and 95% confidence intervals (95% CI) for each variable of interest.

Statistical analyses were performed using Stata SE 15 software (StataCorp, College Station, TX, USA). All statistical tests were two-tailed, and an alpha error of up to 5% was considered acceptable.

### Ethical aspects

This study was approved by the *Faculdade de Medicina* of the *Universidade de São Paulo* Institutional Review Board (CAAE: 17523419.0.0000.0065, #3.492.814). The online survey was open for six months and required eligible candidates to read, understand, and agree to a consent form to participate in the study. The questionnaires were de-identified and anonymized.

## RESULTS

**Figure 1:**
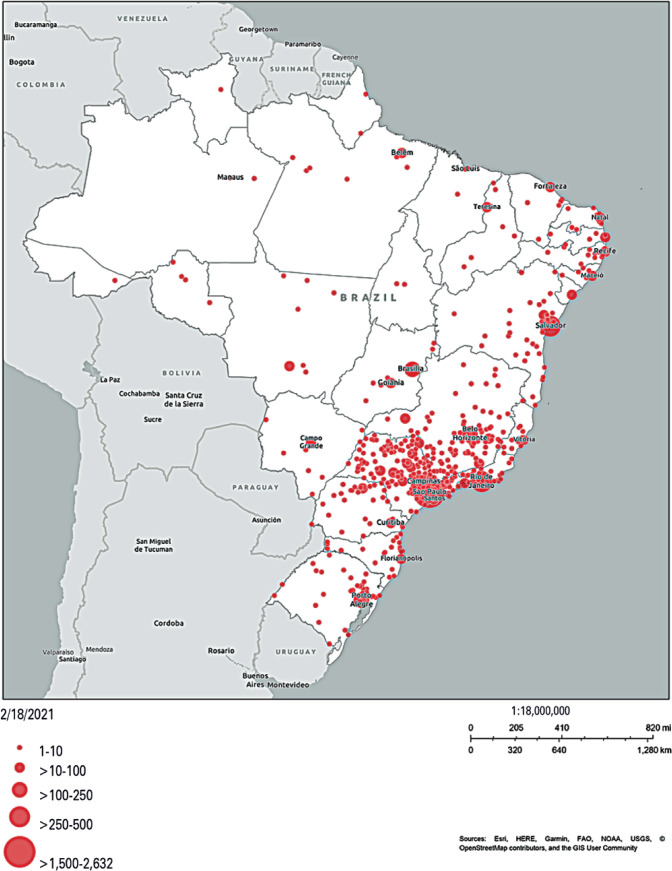
Geographical distribution of participant locations

A total of 7,164 candidates completed the consent form; 347 (5%) did not consent to participate and 124 (2%) provided incomplete answers to our survey. Our final sample included 6,693 participants: 1,332 in the LGBT+ group (20%) and 5,361 in the non-LGBT+ group (80%). There were answers from all the country's macro regions (Figure 1), although there was a noticeable predominance from the southeast.

Overall, the median age was 60 years; 68% were female and 79% were Caucasian (white) (Table 1). Participants had high literacy levels, with 79% having completed university or postgraduate courses. The LGBT+ participants were younger, more frequently male, single, and used the public healthcare system more often. They were also more likely to be living in a rented home (18% *versus* 10%, p<0.001), with an income below the minimum wage (10% *versus* 6%, p<0.001). LGBT+ participants were more likely to report not having anyone to assist them if they became bedridden (22% *versus* 15%, p<0.001).

**Table 1:** Sample characteristics according to LGBT+ group

		Total n=6,693 n (%)	Non-LGBT+ n=5,361 n (%)	LGBT+ n=1,332 n (%)	p value
Frail status (FRAIL Scale)					0.07
	Robust	3,577 (53)	2,901 (54)	676 (51)	
	Prefrail	2,742 (41)	2,170 (40)	572 (43)	
	Frail	374 (6)	290 (5)	84 (6)	
Sex assigned at birth					<0.001
	Male	2,115 (32)	1,343 (25)	772 (58)	
	Female	4,578 (68)	4,018 (75)	560 (42)	
Cisgender		6,444 (96)	5,361 (100)	1,083 (81)	<0.001
Age (years)					<0.001
	50–59	3,257 (49)	2,387 (45)	870 (65)	
	60–69	2,490 (37)	2,112 (39)	378 (28)	
	≥70	946 (14)	862 (16)	84 (6)	
Race/ethnicity					<0.001
	White	5,272 (79)	4,298 (80)	974 (73)	
	Black	357 (5)	301 (6)	56 (4)	
	Other	1,064 (16)	762 (14)	302 (23)	
Literacy					0.24
	College or more	5,272 (79)	4,235 (79)	1,037 (78)	
	High school	1,201 (18)	944 (18)	257 (19)	
	Middle school or less	220 (3)	182 (3)	38 (3)	
Macro-region					<0.001
	Southeast	5,186 (77)	4,241 (79)	945 (71)	
	Southern	458 (7)	350 (7)	108 (8)	
	Central-West	247 (4)	171 (3)	76 (6)	
	Northeast	739 (11)	553 (10)	186 (14)	
	Northern	63 (1)	46 (1)	17 (1)	
Public healthcare system utilization		1,117 (17)	788 (15)	329 (25)	<0.001
Polypharmacy		2,113 (32)	1,680 (31)	433 (33)	0.41
Two or more chronic conditions		1,651 (25)	1,349 (25)	302 (23)	0.06
Depressive Symptoms according to Geriatric Depression Scale		1,986 (30)	1,487 (28)	499 (37)	<0.001
Comorbidities					
	Hypertension	2,483 (37)	2,009 (37)	474 (36)	0.20
	*Diabetes mellitus*	1,028 (15)	827 (15)	201 (15)	0.76
	Cancer	357 (5)	288 (5)	69 (5)	0.78
	Coronary disease	225 (3)	177 (3)	48 (4)	0.58
	Heart failure	201 (3)	156 (3)	45 (3)	0.37
	Cerebrovascular disease	98 (1)	69 (1)	29 (2)	0.03
	Chronic obstructive pulmonary disease	234 (3)	185 (3)	49 (4)	0.69
	Asthma	420 (6)	352 (7)	68 (5)	0.05
	Chronic kidney disease	87 (1)	57 (1)	30 (2)	<0.001

Data are presented as medians (interquartile ranges) for continuous measures and as counts (%) for categorical measures.

In the LGBT+ group, 816 (61%) identified as cisgender homosexual, 199 (15%) as cisgender bisexual, and 68 (5%) as cisgender pansexual or other sexual orientations. A total of 249 (19%) participants identified as transgender or of other genders (29 transgender women, 3 transgender men, 6 *travestis*, and 211 non-binary or other genders).

**Figure 2:**
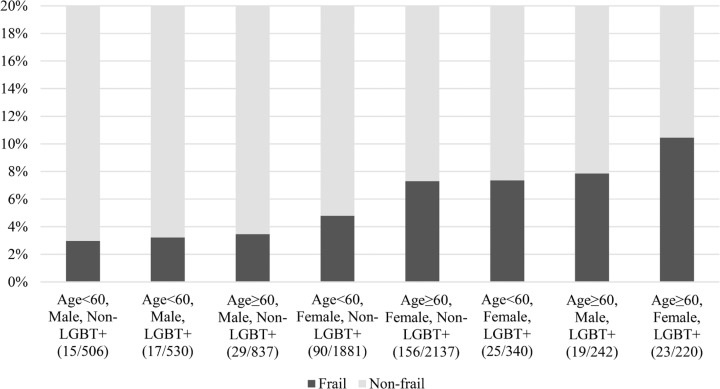
Proportion of frail individuals in each sub-group of the sample

We identified 374 (6%) frail participants in our sample, of which 84 (6%) belonged to the LGBT+ group and 290 (5%) belonged to the non-LGBT+ group. LGBT+ participants were more frequently prefrail or frail than non-LGBT+ participants (49% *versus* 46%, p=0.02). Frailty was more common in LGBT+ females than non-LGBT+ females (9% *versus* 6%, p=0.03), in LGBT+ males aged ≥60 years than non-LGBT+ males of the same age (8% *versus* 3%, p=0.004), and in non-cisgender participants (11% *versus* 5%, p<0.001) (Figure 2).

Multivariable analyses showed that belonging to the LGBT+ group was not independently associated with frailty in the overall sample (OR=1.24, 95%CI=0.95-1.63, p=0.11). However, it was independently associated with frailty in female participants aged ≥50 years (OR=1.52, 95%CI=1.08-2.13, p=0.02) and in male participants aged ≥60 years (OR=2.83, 95%CI=1.41-5.69, p=0.004) (Table 2). Older age, use of the public healthcare system, and several comorbidities were associated with frailty.

**Table 2:** Generalized ordered logistic models examining the association between LGBT+ groups and frailty, according to sex and age

		Prevalence n (%)	Unadjusted odds ratios (95%CI)	Adjusted odds ratios (95%CI)	p value
Overall					
	Age ≥50 years				
	Prefrailty or frailty in LGBT+	656/1,332 (49)	1.14 (1.01-1.29)	1.10 (0.97-1.25)	0.14
	Frailty in LGBT+	84/1,332 (6)	1.18 (0.92-1.51)	1.24 (0.95-1.63)	0.11
	Age = 50-59 years				
	Prefrailty or frailty in LGBT+	426/870 (49)	1.12 (0.96-1.30)	1.04 (0.89-1.23)	0.62
	Frailty in LGBT+	42/870 (5)	1.10 (0.76-1.59)	0.98 (0.66-1.44)	0.92
	Age ≥60 years				
	Prefrailty or frailty in LGBT+	230/462 (50)	1.18 (0.97-1.44)	1.17 (0.96-1.44)	0.13
	Frailty in LGBT+	42/462 (9)	1.51 (1.06-2.14)	1.56 (1.08-2.27)	0.02
Sex assigned at birth = Female					
	Age ≥50 years				
	Prefrailty or frailty in LGBT+	320/560 (57)	1.41 (1.18-1.69)	1.34 (1.12-1.62)	0.002
	Frailty in LGBT+	48/560 (9)	1.44 (1.04-1.99)	1.52 (1.08-2.13)	0.02
	Age = 50-59 years				
	Prefrailty or frailty in LGBT+	194/340 (57)	1.43 (1.13-1.80)	1.31 (1.03-1.67)	0.03
	Frailty in LGBT+	25/340 (7)	1.59 (1.00-2.50)	1.45 (0.89-2.35)	0.13
	Age ≥60 years				
	Prefrailty or frailty in LGBT+	126/220 (57)	1.41 (1.06-1.86)	1.34 (1.00-1.80)	0.05
	Frailty in LGBT+	23/220 (10)	1.48 (0.93-2.35)	1.45 (0.88-2.37)	0.14
Sex assigned at birth = Male					
	Age ≥50 years				
	Prefrailty or frailty in LGBT+	336/772 (44)	1.26 (1.05-1.51)	1.21 (0.99-1.47)	0.06
	Frailty in LGBT+	36/772 (5)	1.44 (0.92-2.26)	1.59 (0.94-2.69)	0.08
	Age = 50-59 years				
	Prefrailty or frailty in LGBT+	232/530 (44)	1.23 (0.96-1.58)	1.15 (0.88-1.49)	0.31
	Frailty in LGBT+	17/530 (3)	1.08 (0.54-2.20)	0.92 (0.42-1.98)	0.82
	Age ≥60 years				
	Prefrailty or frailty in LGBT+	104/242 (43)	1.26 (0.94-1.68)	1.29 (0.95-1.76)	0.10
	Frailty in LGBT+	19/242 (8)	2.37 (1.31-4.31)	2.83 (1.41-5.69)	0.004
Sensitivity analysis					
	Age ≥50 years				
	Prefrailty or frailty in non-cisgender	143/249 (57)	1.58 (1.22-2.03)	1.55 (1.19-2.02)	0.001
	Frailty in non-cisgender	27/249 (11)	2.14 (1.41-3.23)	2.21 (1.42-3.42)	<0.001
	Age = 50-59 years				
	Prefrailty or frailty in non-cisgender	76/134 (57)	1.51 (1.06-2.13)	1.48 (1.03-2.12)	0.03
	Frailty in non-cisgender	9/134 (6)	1.56 (0.76-3.13)	1.43 (0.69-2.96)	0.33
	Age ≥60 years				
	Prefrailty or frailty in non-cisgender	67/115 (58)	1.65 (1.13-2.41)	1.62 (1.09-2.41)	0.02
	Frailty in non-cisgender	18/115 (16)	2.76 (1.64-4.66)	3.04 (1.74-5.33)	<0.001

95%CI: 95% confidence interval; LGBT+: Lesbian, Gay, Bisexual, and Transgender; non-cisgender: transgender, non-binary, and other non-conforming genders.

Finally, in the multivariable sensitivity analysis, we found that the non-cisgender group was independently associated with frailty (odds ratio [OR] =2.21, 95%CI=1.42-3.42, p<0.001). This association was confirmed both among females (OR=2.11, 95%CI=1.23-3.63, p=0.007) and males (OR=2.75, 95%CI=1.30-5.85, p=0.008) (Table 2).

All models were adjusted for age, race/ethnicity, public healthcare utilization, and comorbidities (hypertension, diabetes, cancer, coronary disease, heart failure, chronic obstructive pulmonary disease, asthma, cerebrovascular disease, and chronic kidney disease). The sensitivity analyses were adjusted for sex.

## DISCUSSION

In a large cross-sectional survey, including over 6000 participants, we found that LGBT+ females aged ≥50 and LGBT+ males aged ≥60 years were more likely to be frail than their non-LGBT+ counterparts. Likewise, participants who identified as transgender or other nonconforming genders were more likely to be frail than cisgender participants.

The prevalence of frailty in this study varied from 3% in male non-LGBT+ participants aged 50-59 years to over 10% in female LGBT+ participants aged ≥60 years. The results indicate a lower prevalence than that found in other studies; however, factors such as demographics, frailty definitions, and assessment measures often affect the findings in this field.^(24)^ The ELSI-Brazil study, a populational cohort including more than 8000 participants, reported that frailty occurred in 9% of those aged ≥50 years, 14% of those aged ≥60 years, and 16% of those aged ≥65 years.^(13)^ However, they assessed frailty using the Fried phenotypic criteria, and there was a higher prevalence of multiple chronic conditions in their sample. We preferred to use the FRAIL scale, which can be self-rated and has been validated against the Fried criteria in Brazil,^(19)^ and observed that only one in four of our participants reported two or more chronic conditions.

Moreover, although the ELSI-Brazil Study verified that factors such as less schooling, living without a partner, poor self-rated health, having two or more chronic conditions, and limitations in performing activities of daily living were associated with a higher prevalence of frailty, it did not investigate their association with gender identity or sexual orientation. Evidence of frailty in the context of LGBT+ health is scarce. Baseline data from the Multicenter AIDS Cohort Study found that in a sample of 1,048 men belonging to sexual minorities, 10% were frail and that negative self-perceptions of aging were associated with frailty transitions.^(25)^ But 48% of the cohort was HIV positive, and the study lacked diversity of genders and sexual orientations. Another large cross-sectional study investigating successful aging among LGBT+ older adults surveyed 2,560 LGBT+ adults aged ≥50 years and reported that physical and mental health quality of life was negatively associated with discrimination and chronic conditions.^(26)^ Nonetheless, they did not provide results regarding frailty or comparison with non-LGBT+ adults.

Our study makes a significant contribution to the understanding of health-related difficulties associated with being a member of a gender or sexual minority. In particular, the independent association between being LGBT+ and frailty indicates that this population may experience less healthy aging processes. Frailty is a complex and developing construct, possibly influenced by individual, social, and programmatic factors. Our findings might be partially explained by the fact that conditions such as depression, obesity, and addiction are more common in the LGBT+ population.^(8)^ Furthermore, evidence suggests that LGBT+ persons have less access to healthcare services, which may contribute to lower adherence to health promotion measures, including physical exercise and healthy eating habits.^(27,28)^ Our results appear to confirm these trends since the LGBT+ participants in this study were more reliant on public healthcare, had lower incomes, tended to live in rental homes, and had lower social support.

Another possible explanation for the greater vulnerability to frailty in the LGBT+ group could be related to the "minority stress" theory.^(4)^ This theoretical model suggests that the accumulation of discrimination and stigma experienced by the LGBT+ population, including fear of rejection and internalized homophobia, among others, could make this population more vulnerable to mental illness. Although minority stress was initially thought to be related to mental health issues, evidence suggests that its effects also apply to physical health issues.^(29)^ Transgender and other nonconforming genders likely have additional unstudied characteristics that lead to frailty, and compared to lesbians, gays, and bisexuals, they have considerably worse physical health, disability, depressive symptoms, and stress.^(30)^ A Brazilian study, also conducted online, demonstrated a positive association between internalized homophobia and depression in homosexual men.^(31)^

This study has several limitations. First, this is a cross-sectional study; therefore, causal relationships between gender, sexual orientation, and frailty could not be established. Second, our sample is subject to possible biases intrinsic to snowball sampling strategies and the use of social networks to encourage participation. For instance, the literacy and socioeconomic levels of the sample are above the average Brazilian indicators. Other studies aimed at the LGBT+ population and conducted online also included highly educated individuals, suggesting an inherent bias in this type of research.^(32)^ Thus, the selection of people with higher socioeconomic status may have contributed to the underestimation of the prevalence of frailty in our study. Third, the use of an online questionnaire may have affected the engagement of older contributor age groups. Fourth, gender identity, sexual orientation, healthy aging, and frailty are complex concepts, and there are likely relevant confounders that were not explored in their associations. For example, in addition to the possible difficulty in understanding these concepts, other important factors in the pathophysiology of frailty syndrome, such as protein consumption and strength training, were not considered.

Conversely, there are also important strengths in our work. We included a considerable number of participants (both non-LGBT+ and LGBT+) and examined numerous aspects of their health. Another essential element of our survey was the anonymity of the participants, which enhanced the likelihood of receiving accurate answers concerning subjects generally considered taboo. Furthermore, we obtained a high percentage of complete responses and minimum exclusions for missing data, which indicates the sound quality of the data. Finally, this groundbreaking quantitative study of frailty, gender, and sexual minorities highlights the importance of further research dedicated to aging LGBT+ adults.

## CONCLUSION

In summary, LGBT+ status was found to be independently associated with a higher prevalence of frailty in this study. The LGBT+ community is frequently the victim of intolerance and violence, whether physical or psychological, in a society dominated by hetero-cis-normativity. We hypothesized that such life events might result in social isolation and physical illnesses, including frailty. As frailty represents an additional burden and risk factor for adverse outcomes, it is critical to further investigate its effects on LGBT+ older adults and consider healthy aging interventions specifically tailored for this population.

## Data Availability

All data are available upon request. Requests can be sent to the corresponding author Leonardo Rabelo de Melo.
